# Supplementary material for: Modeling glioblastoma heterogeneity as a dynamic network of cell states
Source: Mol Syst Biol. 2021 Sep 16;17(9):e10105. doi: 10.15252/msb.202010105 (PMC8444284; doi:10.15252/msb.202010105)
Supplement: Supplementary file 5 — Source Data for Figure 3 [file MSB-17-e10105-s001.zip › Figure3A_sourcedata/GSEA_3065/nosigdb_state5.GseaPreranked.1622718435914/FELDMAN_2020_G1.html]

Details for gene set FELDMAN\_2020\_G1[GSEA]

|  || Dataset | state5 |
| Phenotype | NoPhenotypeAvailable |
| Upregulated in class | na\_neg |
| GeneSet | FELDMAN\_2020\_G1 |
| Enrichment Score (ES) | -0.48077473 |
| Normalized Enrichment Score (NES) | -1.7548991 |
| Nominal p-value | 0.0 |
| FDR q-value | 0.003432406 |
| FWER p-Value | 0.093 |
Table: GSEA Results Summary

  

Fig 1: Enrichment plot: FELDMAN\_2020\_G1      
 Profile of the Running ES Score & Positions of GeneSet Members on the Rank Ordered List

  

| PROBE | GENE SYMBOL | GENE\_TITLE | RANK IN GENE LIST | RANK METRIC SCORE | RUNNING ES | CORE ENRICHMENT || 1 | MALAT1 |  |  | 0 | 1.196 | 0.0319 | No |
| 2 | PTN |  |  | 19 | 0.824 | 0.0518 | No |
| 3 | SCRG1 |  |  | 36 | 0.715 | 0.0691 | No |
| 4 | MIAT |  |  | 58 | 0.619 | 0.0832 | No |
| 5 | PRCP |  |  | 103 | 0.523 | 0.0922 | No |
| 6 | SCG2 |  |  | 166 | 0.470 | 0.0977 | No |
| 7 | GGH |  |  | 184 | 0.453 | 0.1078 | No |
| 8 | CTGF |  |  | 199 | 0.440 | 0.1180 | No |
| 9 | ARL6IP1 |  |  | 232 | 0.418 | 0.1255 | No |
| 10 | ORC6 |  |  | 806 | 0.240 | 0.0671 | No |
| 11 | DLG1 |  |  | 924 | 0.221 | 0.0597 | No |
| 12 | ASPM |  |  | 980 | 0.213 | 0.0592 | No |
| 13 | PLP1 |  |  | 1022 | 0.208 | 0.0601 | No |
| 14 | AHNAK2 |  |  | 1427 | 0.159 | 0.0186 | No |
| 15 | CENPF |  |  | 1531 | 0.149 | 0.0109 | No |
| 16 | CYR61 |  |  | 1850 | 0.123 | -0.0218 | No |
| 17 | HNRNPA2B1 |  |  | 1852 | 0.122 | -0.0187 | No |
| 18 | KIF20B |  |  | 1962 | 0.114 | -0.0279 | No |
| 19 | IGFBP3 |  |  | 2037 | 0.109 | -0.0334 | No |
| 20 | HAT1 |  |  | 2459 | 0.082 | -0.0789 | No |
| 21 | ATAD2 |  |  | 2545 | 0.077 | -0.0865 | No |
| 22 | RNASEH2A |  |  | 2560 | 0.076 | -0.0860 | No |
| 23 | AURKB |  |  | 2853 | 0.060 | -0.1175 | No |
| 24 | CKAP2L |  |  | 2979 | 0.053 | -0.1302 | No |
| 25 | SRSF7 |  |  | 3126 | 0.046 | -0.1455 | No |
| 26 | NCAPG |  |  | 3194 | 0.041 | -0.1520 | No |
| 27 | IGFBP5 |  |  | 3230 | 0.040 | -0.1549 | No |
| 28 | TPST2 |  |  | 3303 | 0.036 | -0.1621 | No |
| 29 | SMC2 |  |  | 3374 | 0.032 | -0.1691 | No |
| 30 | ESCO2 |  |  | 3672 | 0.021 | -0.2022 | No |
| 31 | GTSE1 |  |  | 3738 | 0.018 | -0.2090 | No |
| 32 | DLGAP5 |  |  | 3907 | 0.011 | -0.2278 | No |
| 33 | NDC80 |  |  | 4034 | 0.006 | -0.2419 | No |
| 34 | PRC1 |  |  | 4241 | -0.003 | -0.2651 | No |
| 35 | NUF2 |  |  | 4628 | -0.019 | -0.3083 | No |
| 36 | CENPK |  |  | 4675 | -0.021 | -0.3129 | No |
| 37 | CDK1 |  |  | 4766 | -0.025 | -0.3224 | No |
| 38 | CENPE |  |  | 4787 | -0.025 | -0.3240 | No |
| 39 | TROAP |  |  | 4869 | -0.028 | -0.3324 | No |
| 40 | DDX39A |  |  | 4948 | -0.031 | -0.3404 | No |
| 41 | MCM7 |  |  | 4986 | -0.032 | -0.3438 | No |
| 42 | GINS2 |  |  | 5024 | -0.034 | -0.3471 | No |
| 43 | CCNB1 |  |  | 5111 | -0.037 | -0.3558 | No |
| 44 | SPC25 |  |  | 5312 | -0.045 | -0.3772 | No |
| 45 | CCNB2 |  |  | 5382 | -0.048 | -0.3838 | No |
| 46 | NUSAP1 |  |  | 5389 | -0.048 | -0.3832 | No |
| 47 | BUB3 |  |  | 5596 | -0.057 | -0.4050 | No |
| 48 | CLSPN |  |  | 5614 | -0.058 | -0.4053 | No |
| 49 | CCNA2 |  |  | 5671 | -0.061 | -0.4100 | No |
| 50 | TOP2A |  |  | 5722 | -0.063 | -0.4140 | No |
| 51 | CDCA3 |  |  | 5726 | -0.063 | -0.4127 | No |
| 52 | KIF22 |  |  | 5758 | -0.064 | -0.4145 | No |
| 53 | KPNA2 |  |  | 5811 | -0.066 | -0.4186 | No |
| 54 | KNSTRN |  |  | 5923 | -0.070 | -0.4293 | No |
| 55 | EXOSC8 |  |  | 6020 | -0.075 | -0.4382 | No |
| 56 | MKI67 |  |  | 6211 | -0.086 | -0.4574 | No |
| 57 | RRM1 |  |  | 6298 | -0.090 | -0.4647 | No |
| 58 | RRM2 |  |  | 6308 | -0.091 | -0.4633 | No |
| 59 | CENPM |  |  | 6368 | -0.093 | -0.4675 | No |
| 60 | CKAP2 |  |  | 6372 | -0.094 | -0.4653 | No |
| 61 | CENPA |  |  | 6374 | -0.094 | -0.4630 | No |
| 62 | ZWINT |  |  | 6453 | -0.099 | -0.4692 | No |
| 63 | DNMT1 |  |  | 6455 | -0.099 | -0.4666 | No |
| 64 | SMC4 |  |  | 6517 | -0.102 | -0.4708 | No |
| 65 | KIF4A |  |  | 6529 | -0.103 | -0.4693 | No |
| 66 | DBF4 |  |  | 6572 | -0.106 | -0.4712 | No |
| 67 | PLK1 |  |  | 6581 | -0.107 | -0.4693 | No |
| 68 | MAD2L1 |  |  | 6600 | -0.108 | -0.4684 | No |
| 69 | HNRNPDL |  |  | 6625 | -0.109 | -0.4682 | No |
| 70 | LMNB1 |  |  | 6638 | -0.110 | -0.4666 | No |
| 71 | VRK1 |  |  | 6651 | -0.111 | -0.4650 | No |
| 72 | ANP32E |  |  | 6655 | -0.111 | -0.4624 | No |
| 73 | CEP55 |  |  | 6700 | -0.114 | -0.4644 | No |
| 74 | DEPDC1 |  |  | 6723 | -0.115 | -0.4638 | No |
| 75 | CDCA2 |  |  | 6730 | -0.116 | -0.4614 | No |
| 76 | KIF23 |  |  | 6764 | -0.118 | -0.4619 | No |
| 77 | FBXO5 |  |  | 6775 | -0.119 | -0.4599 | No |
| 78 | CCDC34 |  |  | 6826 | -0.123 | -0.4623 | No |
| 79 | ANLN |  |  | 6829 | -0.123 | -0.4592 | No |
| 80 | HSP90AA1 |  |  | 6962 | -0.134 | -0.4706 | No |
| 81 | CDC20 |  |  | 6985 | -0.136 | -0.4695 | No |
| 82 | AURKA |  |  | 6990 | -0.136 | -0.4663 | No |
| 83 | PBK |  |  | 7010 | -0.138 | -0.4648 | No |
| 84 | DNAJC9 |  |  | 7043 | -0.141 | -0.4646 | No |
| 85 | MIS18A |  |  | 7115 | -0.147 | -0.4688 | No |
| 86 | CENPW |  |  | 7116 | -0.147 | -0.4649 | No |
| 87 | FEN1 |  |  | 7201 | -0.155 | -0.4702 | No |
| 88 | GMNN |  |  | 7224 | -0.158 | -0.4685 | No |
| 89 | TMPO |  |  | 7296 | -0.167 | -0.4721 | No |
| 90 | CENPH |  |  | 7329 | -0.171 | -0.4712 | No |
| 91 | TK1 |  |  | 7377 | -0.178 | -0.4717 | No |
| 92 | USP1 |  |  | 7398 | -0.181 | -0.4692 | No |
| 93 | PSIP1 |  |  | 7460 | -0.187 | -0.4711 | No |
| 94 | CENPN |  |  | 7528 | -0.194 | -0.4735 | No |
| 95 | CMC2 |  |  | 7539 | -0.196 | -0.4694 | No |
| 96 | TPX2 |  |  | 7549 | -0.197 | -0.4651 | No |
| 97 | UBE2T |  |  | 7561 | -0.199 | -0.4611 | No |
| 98 | BIRC5 |  |  | 7596 | -0.204 | -0.4595 | No |
| 99 | RAD21 |  |  | 7785 | -0.231 | -0.4746 | Yes |
| 100 | MZT1 |  |  | 7838 | -0.242 | -0.4741 | Yes |
| 101 | PDLIM7 |  |  | 7864 | -0.247 | -0.4703 | Yes |
| 102 | CENPU |  |  | 7871 | -0.248 | -0.4644 | Yes |
| 103 | DHFR |  |  | 7878 | -0.250 | -0.4584 | Yes |
| 104 | H2AFX |  |  | 7965 | -0.269 | -0.4609 | Yes |
| 105 | CKS2 |  |  | 7972 | -0.270 | -0.4544 | Yes |
| 106 | HMGB2 |  |  | 7973 | -0.271 | -0.4472 | Yes |
| 107 | HIST1H1C |  |  | 7978 | -0.271 | -0.4404 | Yes |
| 108 | MAP3K7CL |  |  | 8063 | -0.288 | -0.4422 | Yes |
| 109 | DTYMK |  |  | 8075 | -0.292 | -0.4357 | Yes |
| 110 | CDKN3 |  |  | 8079 | -0.293 | -0.4282 | Yes |
| 111 | PCNA |  |  | 8082 | -0.293 | -0.4206 | Yes |
| 112 | PHF19 |  |  | 8093 | -0.295 | -0.4139 | Yes |
| 113 | DUT |  |  | 8118 | -0.300 | -0.4086 | Yes |
| 114 | TMEM106C |  |  | 8146 | -0.308 | -0.4034 | Yes |
| 115 | HIST1H1E |  |  | 8176 | -0.320 | -0.3982 | Yes |
| 116 | ILF2 |  |  | 8220 | -0.331 | -0.3942 | Yes |
| 117 | CKS1B |  |  | 8241 | -0.336 | -0.3875 | Yes |
| 118 | LSM5 |  |  | 8242 | -0.337 | -0.3786 | Yes |
| 119 | UBE2C |  |  | 8278 | -0.348 | -0.3732 | Yes |
| 120 | TUBB4B |  |  | 8356 | -0.371 | -0.3721 | Yes |
| 121 | NUDT1 |  |  | 8359 | -0.373 | -0.3623 | Yes |
| 122 | HMGB3 |  |  | 8427 | -0.399 | -0.3593 | Yes |
| 123 | HIST1H1B |  |  | 8437 | -0.402 | -0.3496 | Yes |
| 124 | PTTG1 |  |  | 8446 | -0.406 | -0.3396 | Yes |
| 125 | NUCKS1 |  |  | 8502 | -0.435 | -0.3343 | Yes |
| 126 | SNRPD1 |  |  | 8537 | -0.451 | -0.3261 | Yes |
| 127 | C12orf75 |  |  | 8546 | -0.456 | -0.3148 | Yes |
| 128 | RAN |  |  | 8555 | -0.463 | -0.3034 | Yes |
| 129 | RPA3 |  |  | 8572 | -0.471 | -0.2926 | Yes |
| 130 | PTGES3 |  |  | 8587 | -0.479 | -0.2814 | Yes |
| 131 | TYMS |  |  | 8599 | -0.488 | -0.2697 | Yes |
| 132 | HMGN2 |  |  | 8608 | -0.496 | -0.2573 | Yes |
| 133 | CYCS |  |  | 8640 | -0.519 | -0.2470 | Yes |
| 134 | HMGB1 |  |  | 8653 | -0.527 | -0.2343 | Yes |
| 135 | SIVA1 |  |  | 8654 | -0.529 | -0.2202 | Yes |
| 136 | ANP32B |  |  | 8680 | -0.545 | -0.2085 | Yes |
| 137 | SNRPB |  |  | 8683 | -0.547 | -0.1941 | Yes |
| 138 | H2AFV |  |  | 8693 | -0.554 | -0.1804 | Yes |
| 139 | CALM2 |  |  | 8744 | -0.600 | -0.1700 | Yes |
| 140 | DEK |  |  | 8770 | -0.627 | -0.1561 | Yes |
| 141 | H2AFZ |  |  | 8774 | -0.630 | -0.1396 | Yes |
| 142 | STMN1 |  |  | 8783 | -0.638 | -0.1235 | Yes |
| 143 | LSM3 |  |  | 8803 | -0.655 | -0.1082 | Yes |
| 144 | RANBP1 |  |  | 8819 | -0.669 | -0.0921 | Yes |
| 145 | HIST1H4C |  |  | 8850 | -0.710 | -0.0766 | Yes |
| 146 | PTMA |  |  | 8894 | -0.764 | -0.0611 | Yes |
| 147 | UBE2S |  |  | 8901 | -0.772 | -0.0412 | Yes |
| 148 | S100B |  |  | 8908 | -0.784 | -0.0209 | Yes |
| 149 | TUBA1B |  |  | 8980 | -1.103 | 0.0005 | Yes |
Table: GSEA details [plain text format]

  

Fig 2: FELDMAN\_2020\_G1: Random ES distribution      
 Gene set null distribution of ES for **FELDMAN\_2020\_G1**
